# DNA-methylation profiling distinguishes malignant melanomas from benign nevi

**DOI:** 10.1111/j.1755-148X.2011.00828.x

**Published:** 2011-01-21

**Authors:** Kathleen Conway, Sharon N Edmiston, Zakaria S Khondker, Pamela A Groben, Xin Zhou, Haitao Chu, Pei Fen Kuan, Honglin Hao, Craig Carson, Marianne Berwick, David W Olilla, Nancy E Thomas

**Affiliations:** 1 Department of Epidemiology, School of Public Health, University of North CarolinaChapel Hill, NC, USA; 2 Lineberger Comprehensive Cancer Center, University of North CarolinaChapel Hill, NC, USA; 3 Department of Biostatistics, School of Public Health, University of North CarolinaChapel Hill, NC, USA; 4 Department of Pathology and Laboratory Medicine, School of Medicine, University of North CarolinaChapel Hill, NC, USA; 5 Department of Biostatistics, University of MinnesotaMinneapolis, MN, USA; 6 Department of Dermatology, School of Medicine, University of North CarolinaChapel Hill, NC, USA; 7 Department of Medicine, University of New MexicoAlbuquerque, NM, USA; 8 Department of Surgery, School of Medicine, University of North CarolinaChapel Hill, NC, USA

**Keywords:** melanoma, nevi, methylation profiling, diagnostic markers

## Abstract

DNA methylation, an epigenetic alteration typically occurring early in cancer development, could aid in the molecular diagnosis of melanoma. We determined technical feasibility for high-throughput DNA-methylation array-based profiling using formalin-fixed paraffin-embedded tissues for selection of candidate DNA-methylation differences between melanomas and nevi. Promoter methylation was evaluated in 27 common benign nevi and 22 primary invasive melanomas using a 1505 CpG site microarray. Unsupervised hierarchical clustering distinguished melanomas from nevi; 26 CpG sites in 22 genes were identified with significantly different methylation levels between melanomas and nevi after adjustment for age, sex, and multiple comparisons and with β-value differences of ≥0.2. Prediction analysis for microarrays identified 12 CpG loci that were highly predictive of melanoma, with area under the receiver operating characteristic curves of >0.95. Of our panel of 22 genes, 14 were statistically significant in an independent sample set of 29 nevi (including dysplastic nevi) and 25 primary invasive melanomas after adjustment for age, sex, and multiple comparisons. This first report of a DNA-methylation signature discriminating melanomas from nevi indicates that DNA methylation appears promising as an additional tool for enhancing melanoma diagnosis.

**Significance**

Early diagnosis significantly improves melanoma survival; yet the histologic diagnosis of melanoma can be challenging even for experienced pathologists. We established the feasibility of high-throughput DNA-methylation assays on formalin-fixed paraffin-embedded melanocytic tissues, as are typically prepared in hospital and community-based dermatologic practices. Our study is the first to discover a multilocus DNA-methylation signature that discriminates melanomas from nevi, indicating that DNA methylation holds promise for molecular diagnosis of melanoma. In addition, knowledge of DNA-methylation differences between melanomas and nevi could improve our understanding of melanomagenesis, identify new therapeutic targets, and provide the thrust for detection of occult melanoma in lymph nodes and blood for staging and monitoring patients.

For melanoma, there is a pronounced survival difference between localized and metastatic disease (98% and 15–62% 5-year survival, respectively) (ACS, 2010), making it imperative to diagnose melanoma early. However, histologic diagnosis of melanocytic lesions can be problematic because a single melanocytic lesion may exhibit conflicting diagnostic criteria, making a definitive diagnosis of either a benign nevus or melanoma difficult. One study reported 15% discordance in the diagnosis of melanocytic lesions (Shoo et al., 2010). An earlier study of over 1000 melanocytic lesions reported that an expert panel found a false positive rate of 14%, misclassifying benign lesions as invasive melanoma, and a false negative rate of 17%, misclassifying malignant melanoma as benign (Veenhuizen et al., 1997). In fact, many nevi, especially atypical or dysplastic nevi, are difficult to distinguish from melanoma, even by expert pathologists (Farmer et al., 1996).

Studies suggest that DNA methylation may provide a valuable tool, in conjunction with histopathology, for the molecular diagnostics of melanoma. DNA methylation is an epigenetic chemical modification that does not alter the sequence code, but can be heritable, and is involved in the regulation of gene expression (Plass, 2002). The most common methylation site in mammals is a cytosine located next to a guanosine (CpG). Clusters of CpGs, referred to as islands, are found in the 5′ regulatory and promoter regions of genes (Antequera and Bird, 1993). Hypermethylation of CpG islands in promoter regions is a common mechanism of tumor suppressor gene silencing in cancer (Herman and Baylin, 2003). Because aberrant promoter methylation with silencing of tumor suppressor genes has been shown to occur widely in human melanomas (Furuta et al., 2004; Hoon et al., 2004) and in histologically premalignant lesions associated with a variety of cancer types (Fackler et al., 2003), methylation appears promising as an early diagnostic marker for melanoma.

In this study, we investigated high-throughput array-based DNA-methylation profiling using the Illumina GoldenGate methylation Cancer Panel I array, which is designed to detect methylation at 1505 CpG sites in the promoters and regulatory regions of 807 cancer-related genes, to examine technical feasibility as to whether profiling could be accomplished on formalin-fixed tissues and ‘proof of principle’ that DNA methylations could distinguish melanomas from nevi. The Cancer Panel I methylation array was previously validated in side-by-side comparisons with methylation-specific PCR and bisulfite sequencing and showed strong correlations with both methods (Bibikova et al., 2006). After optimizing conditions for performance of the array, we evaluated array reproducibility and correlation between fresh versus formalin-fixed specimens. In addition, the effect of intermixture of melanocytic with non-melanocytic DNA on methylation profiles was examined to estimate tumor purity necessary for target tissue profiling. Moreover, we compared the methylation profiles of primary melanomas to benign nevi.

We initially tested a range of bisulfite-treated DNA quantities (100–500 ng) in the Illumina GoldenGate methylation Cancer Panel I array, and identified 200 ng non-fixed or 250 ng of formalin-fixed bisulfite-treated DNA as the minimum quantity needed to successfully perform array profiling. Such quantities were recoverable from the FFPE melanocytic tissues in this study.

We found very high reproducibility between non-fixed cell lines and the same lines that had undergone the FFPE process. Cell lines were pelleted, formalin-fixed, and paraffin-embedded just as tissue is in the clinical setting to create FFPE-processed equivalents for cell lines. Shown in Figure S1A are replicate methylation array profiles of non-formalin-fixed MCF-7 breast tumor cell DNA, formalin-fixed DNA from the Mel-505 melanoma cell line, as well as methylation profiles from non-fixed versus FFPE Mel-505 DNA. Each of these array replicates produced highly correlated methylation profiles, showing r^2^ values of ≥0.98. Methylation analysis on eight non-fixed cell line replicates, 20 additional non-fixed and FFPE melanoma cell line pairs, and 14 FFPE melanoma cell line replicates also yielded highly concordant methylation patterns with a mean *r*^2^ of 0.98, 0.98, and 0.97, respectively (not shown). These results confirmed that the Illumina GoldenGate Cancer Panel I methylation array was highly reproducible, and formalin fixation of the DNA template did not alter the methylation profile.

In mixing experiments, we estimated the proportion of melanoma cell line Mel-505 DNA that must be present in a cancer/normal DNA mixture in order for the melanoma methylation profile to remain evident. In Figure S1B, Mel-505 cell line DNA was diluted with increasing proportions (from 0 to 50%) of DNA from normal peripheral blood leukocytes (PBLs), and each mixture was plotted against the profile for pure (100%) Mel-505 cell line DNA. The Mel-505 cell line profile was evident even after dilution with up to 30% PBL DNA (70% Mel-505/30% PBL mixture) (r^*2*^ = 0.89), indicating that a moderate level of contamination of melanocytic DNA by normal DNA did not significantly disrupt the melanoma methylation pattern. Similarly, FFPE melanoma DNA mixed with increasing proportions of FFPE normal skin DNA maintained melanoma methylation profiles when 70% (r^2^ = 0.84) to 80% (r^2^ = 0.91) melanoma DNA was present (Figure S1C).

In order to compare methylation patterns in nevi and melanomas, 22 primary invasive melanomas and 27 benign nevi (sample set #1) underwent DNA-methylation profiling using the Illumina GoldenGate Cancer Panel I and passed filtering criteria. For all comparisons of Illumina methylation array results, we removed 68 probes that corresponded to CpG sites on the X chromosome and 410 probes that were reported to contain a SNP or repeat (Byun et al., 2009), thus making them unreliable in some samples. Additionally, β-values with a detection P-value >10^−5^ were considered unreliable and set as missing data points (Marsit et al., 2009); using this criterion, two nevus samples with more than 25% missing β-values as well as 39 CpG loci with β-values missing in more than 20% of samples were excluded from analysis. The final data set consisted of 22 melanomas of a variety of histologic subtypes and Breslow thicknesses (range 0.59–10.0 mm) and 27 common benign nevi (Table S1) with DNA-methylation profiling at 988 CpG loci within 646 genes.

Unsupervised hierarchical clustering was used to compare β methylation values at CpG loci between melanomas and nevi. Clustering produced a clear separation of melanomas from nevi, with at least two major clusters each of melanomas and nevi identified (Figure 1A), suggesting that the methylation signature of melanomas is fundamentally distinct from that of the nevi included in this study. Using class comparison analyses, 168 CpG sites were identified that differed significantly (with adjusted P-values of <0.05) between melanomas and nevi after Bonferroni correction for multiple comparisons (Table S2). Seventy-five of these CpG sites (in 63 genes) differed by ≥0.2β.

**Figure 1:**
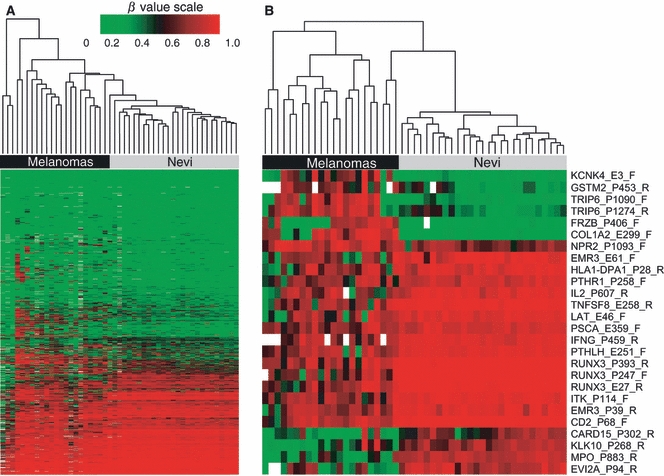
Hierarchical clustering of methylation β-values using the Illumina GoldenGate Cancer Panel I array in FFPE nevi and melanomas from sample set #1. DNA-methylation profiles for 22 melanomas and 27 nevi are shown. Columns represent tissue samples; rows represent CpG loci. Methylation level (β) from 0 (green/unmethylated) to 1 (red/highly methylated). Missing values are shown in white. (A) Unsupervised clustering based on 988 CpG sites in 646 genes after filtering (see Methods); probes included autosomal loci and those with a detection P-value of <0.05. (B) Clusters based on the 26 CpG sites showing significantly different methylation β levels between nevi and melanomas after adjustment for age and sex and Bonferroni correction for multiple comparisons. The upper portion of the heatmap shows seven CpG loci in six genes exhibiting hypermethylation, and the lower portion shows 19 CpG loci in 16 genes exhibiting hypomethylation in melanomas compared with nevi.

After further adjustment for patient's age and sex, we identified a total of 26 CpG loci in 22 genes that differed significantly between melanomas and nevi, including 19 CpG loci that were significantly hypomethylated and 7 CpG loci that were significantly hypermethylated in melanoma compared to nevi (Table 1). The heatmap based on unsupervised clustering of the 26 differentially methylated CpG loci in melanomas and nevi is shown in Figure 1B. The genes with loci showing relative hypermethylation in melanomas compared with nevi were *KCNK4*, *GSTM2*, *TRIP6* (2 CpG sites), *FRZB*, *COL1A2*, and *NPR2*. The genes with relative hypomethylated loci in melanoma were *CARD15/NOD2*, *KLK10*, *MPO*, *EVI2A*, *EMR3* (two sites), *HLA-DPA1*, *PTHR1*, *IL2*, *TNFSF8*, *LAT*, *PSCA*, *IFNG*, *PTHLH*, *RUNX3* (three CpG sites), *ITK*, and *CD2*. *ITK* had a second CpG locus that was significantly different between melanomas and nevi but the β-value difference was <0.2. Five of seven (71%) hypermethylated CpG loci and 13/19 (68%) of hypomethylated loci were located within gene promoters (according to Illumina annotation). Six of the seven (86%) CpG loci exhibiting hypermethylation in melanomas were located within CpG islands, while only 2/19 (11%) CpG loci showing hypomethylation were within CpG islands.

**Table 1:** Twenty-six CpG loci identified in sample set #1 exhibiting significant promoter methylation differences and β-value differences ≥0.2 between primary melanomas and nevi after adjustment for age, sex, and Bonferroni correction for multiple comparisons

			Sample set 1					Sample set 2
				
Gene symbol	CpG probe	Gene description	Nevus mean β	Melanoma mean β	P-value^1^	Mean Δβ	AUC	AUC
Hypermethylated in melanomas compared with nevi (n = 7)								
*COL1A2*	E299_F	Collagen, type I, alpha 2	0.065	0.623	4.07 × 10^−5^	−0.558	0.9007	0.6248
*FRZB*	P406_F	Frizzled-related protein	0.039	0.374	1.45 × 10^−2^	−0.334	0.8986	0.7613***
*GSTM2*	P453_R	Glutathione S-transferase M2	0.145	0.590	6.31 × 10^−3^	−0.445	0.9186	0.5731
*KCNK4*	E3_F	Potassium channel, subfamily K, member 4	0.089	0.479	2.62 × 10^−3^	−0.390	0.9057	0.7600
*NPR2*	P1093_F	Natriuretic peptide receptor B/guanylate cyclase B	0.564	0.833	1.78 × 10^−2^	−0.269	0.8434	0.6581
*TRIP6*	P1090_F	Thyroid hormone receptor interactor 6	0.099	0.667	6.30 × 10^−5^	−0.568	0.8518	0.7186
*TRIP6*	P1274_R	Thyroid hormone receptor interactor 6	0.187	0.705	2.71 × 10^−3^	−0.518	0.8704	0.5945
Hypomethylated in melanomas compared with nevi (n = 19)								
*CARD15/NOD2*	P302_R	Caspase recruitment domain family, member 15	0.560	0.114	3.09 × 10^−2^	0.446	0.8754	0.7874**
*CD2*	P68_F	CD2 molecule	0.961	0.735	1.26 × 10^−7^	0.227	0.9983	0.8414*
*EMR3*	E61_F	EGF-like module-containing mucin-like hormone receptor-like 3	0.921	0.485	1.35 × 10^−3^	0.436	0.9242	0.9503*
*EMR3*	P39_R	EGF-like module-containing mucin-like hormone receptor-like 3	0.920	0.633	2.00 × 10^−3^	0.286	0.9259	0.8391*
*EV12A*	P94_R	Ecotropic viral integration site 2A	0.783	0.265	1.35 × 10^−3^	0.518	0.9592	0.9669*
*HLA-DPA1*	P28_R	Major histocompatibility complex, class II, DP alpha 1	0.902	0.565	3.32 × 10^−2^	0.337	0.9191	0.8400*
*IFNG*	P459_R	Interferon gamma	0.915	0.634	7.91 × 10^−9^	0.281	0.9630	0.9670*
*IL2*	P607_R	Interleukin 2	0.887	0.590	8.97 × 10^−3^	0.297	0.9489	0.8801*
*ITK*	P114_F	IL2-inducible T-cell kinase	0.924	0.632	2.68 × 10^−6^	0.292	0.9663	0.9076*
*KLK10*	P268_R	Kallikrein-related peptidase 10	0.673	0.257	4.38 × 10^−2^	0.417	0.9040	0.6997
*LAT*	E46_F	Linker for activation of T cells	0.895	0.540	1.75 × 10^−2^	0.355	0.9646	0.8328*
*MPO*	P883_R	Myeloperoxidase	0.759	0.165	2.39 × 10^−6^	0.593	0.9983	0.8908*
*PSCA*	E359_F	Prostate stem cell antigen	0.859	0.658	5.17 × 10^−3^	0.201	0.8788	0.6028
*PTHLH*	E251_R	Parathyroid hormone-like hormone	0.923	0.602	5.80 × 10^−6^	0.322	0.9933	0.8497*
*PTHR1*	P258_F	Parathyroid hormone receptor 1	0.849	0.593	4.56 × 10^−3^	0.256	0.8889	0.5848
*RUNX3*	P393_R	Runt-related transcription factor 3	0.967	0.735	3.26 × 10^−8^	0.232	1.0000	0.9200*
*RUNX3*	E27_R	Runt-related transcription factor 3	0.960	0.665	6.53 × 10^−8^	0.296	0.9983	0.9393*
*RUNX3*	P247_F	Runt-related transcription factor 3	0.964	0.627	1.06 × 10^−8^	0.337	1.0000	0.9324*
*TNFSF8*	E258_R	Tumor necrosis factor superfamily, member 8	0.952	0.599	1.64 × 10^−7^	0.353	0.9949	0.9462*

1 P-value, nevus mean β, and melanoma mean β were each adjusted for age, sex, and multiple comparisons using Bonferroni correction.

AUC; area under the ROC curve.

P-value significant for difference between melanomas and nevi in sample set #2 at the 0.05* or 0.0505** level after adjustment for age, sex, and multiple comparisons using Bonferroni correction. ***FRZB_P406_F (AUC of 0.7613) was not significantly differentially methylated in sample set #2; however, a different CpG site within the *FRZB* gene (FRZB_E186_R) was with AUC of 0.8648.

Prediction analysis for microarrays (PAM) was carried out using 988 CpG sites to assess the classification of melanoma and nevus samples by the method of nearest shrunken centroids (Tibshirani et al., 2002). The PAM algorithm automatically identifies CpG loci that contribute most to the melanoma classification. Using 10-fold cross-validation to train the classifier, the optimal shrinkage threshold was chosen to be 4.28 with 12 CpG loci required for optimal classification. This approach yielded a zero cross-validation error, with no misclassification. The 12 CpG loci identified by PAM analysis that provided the most accurate prediction of melanoma were RUNX3_P393_R, RUNX3_P247_F, RUNX3_E27_R, COL1A2_E299_F, MPO_P883_R, TNFSF8_E258_R, CD2_P68_F, EVI2A_P94_R, OSM_P188_F, ITK_P114_F, FRZB_P406_F, and ITK_E166_R.

The box plots shown in Figure 2A display the distribution of β-values in nevi and melanomas for the 12 CpG sites that are highly predictive of melanoma as determined by PAM analysis. For most CpG loci showing hypomethylation in melanomas, mean methylation β-values were uniformly very high (β nearly 1.0) in nevi, and this methylation was diminished to varying degrees in melanomas. Among the CpG loci exhibiting hypermethylation in melanomas, FRZB_P406_F and COL1A2_E299_F were relatively unmethylated in nevi, having mean β-values near 0.1, but showed considerably higher methylation in many melanomas, with mean β-values between 0.6 and 0.7.

**Figure 2:**
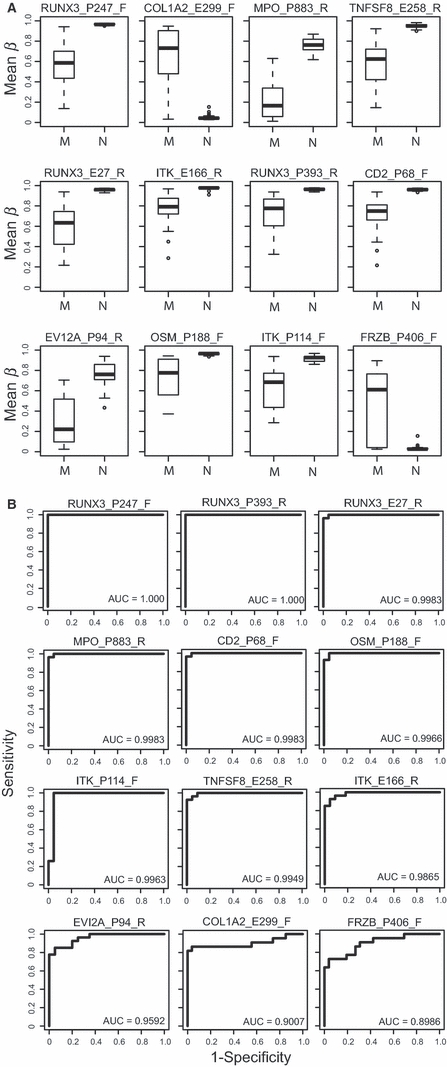
CpG loci that predict melanoma identified by PAM analysis in sample set #1. (A) Box plots of methylation β levels in the 12 CpG loci identified by PAM analysis as being predictive of melanoma. CpG loci shown differed by ≥0.2 mean β between melanomas (M) and nevi (N), except for ITK_P114_F. Each box plot shows the median β-value (dark bar within box), the interquartile range (IQR = Q3−Q1) (outer boundaries of box). The whiskers (broken line) cover (Q1−1.5IQR, Q3 + 1.5IQR). Additional information on mean β-values for nevi and melanomas, differences in mean β-values, and P-values adjusted for age, sex, and multiple comparisons using Bonferroni correction are given in Table 1. (B) ROC curves showing the sensitivity versus 1-specificity of the 12 CpG loci identified by PAM that predict melanoma. Sensitivity, the true positive rate, is shown along the *y*-axis, while 1-specificity, or the false positive rate, is shown along the *x*-axis. The calculated AUC is given for each plot in rank order beginning with highest AUC.

Receiver operating characteristic (ROC) curves were generated showing the sensitivity versus 1-specificity of the 12 CpG loci identified by PAM analysis, which differentiate melanomas from nevi (Figure 2B). The areas under the ROC curves (AUCs) ranged from 0.89 to 0.90 for the two hypermethylated loci to 0.96–1.00 for the ten hypomethylated loci. In particular, two of the RUNX3 probes (RUNX3_P247_F and RUNX3_P393_R) exhibited 100% sensitivity and 100% specificity in identifying melanomas.

We explored the major functions of the 22 differentially methylated genes (with 26 CpG sites) that most significantly distinguished melanomas from nevi and found associations with several major functions and pathways including apoptosis, cell cycle, proliferation, cell adhesion, cell communication and signaling, and immune response (Table 1). Because the genes were preselected as part of a cancer panel, only a limited evaluation could be carried out of functional pathways using the DAVID Bioinformatics Resources 6.7 (http://david.abcc.ncifcrf.gov/home.jsp). Of interest, half (11 of 22) of the genes possessed putative immune-related functions, including roles in T-cell signaling and/or natural killer cell cytotoxicity (*IFNG*, *IL2*, *ITK*, *LAT*, *CD2*, *TNFSF8*, *HLA-DPA1*), myeloid–myeloid cell interactions (*EMR3*), neutrophil microbicidal activity (*MPO*), innate immunity (*CARD15/NOD2*), and NF-κB activation (*TRIP6, CARD15/NOD2*). Three genes function in thyroid (*TRIP6*) or parathyroid (*PTHLH*, *PTHR1*) hormonal regulation. Several others are tumor suppressor genes or have previously described or suspected roles in cancer cell growth, cell adhesion, or apoptosis (*RUNX3*, *FRZB*, *TNFSF8*, *KLK10*, *PSCA*, *COL1A2*). Others have been noted as targets of the polycomb repressor complex in stem cells (*RUNX3* and *PTHLH* from the 26 CpG site list, and *RARA*, *HOXA11*, *GABRA5*, *LOX*, and *PGR* from the 75 CpG site list) (Lee et al., 2006a; O'Riain et al., 2009).

A second independent set of 25 melanomas and 29 nevi (sample set #2) were tested for DNA methylation using the Illumina GoldenGate Cancer Panel I and passed filtering criteria (Table S1). The melanomas were of a variety of histologic subtypes and ranged in Breslow thickness from 0.42 to 10.75 mm. The majority of nevi (21 of 29) had varying degrees of histologic atypia. Of our panel of 22 genes identified through analysis of sample set #1, 14 were also statistically significant for differential methylation in sample set #2 after Bonferroni correction and adjustment for age and sex (Table 1). These genes were *CARD15, CD2, EMR3* (two CpG loci)*, EVI2A, FRZB, HLA-DPA1, IFNG, IL2, ITK, LAT, MPO, PTHLH, RUNX3* (three CpG loci), and *TNFSF8*. It should be noted that the FRZB_E186 CpG locus rather than FRZB_P406 was significantly differentially methylated in sample set #2. The AUCs for CpG sites within these genes remained high in sample set #2, ranging from 0.79 to 0.97.

We found that high-throughput DNA-methylation array profiling was feasible for FFPE melanoctyic lesions and approximately 250 ng of bisulfite-treated DNA and 70% tumor DNA purity were necessary to achieve methylation array results representative of melanocytic target DNA. We identified 168 differentially methylated CpG loci that significantly distinguish melanomas from nevi after adjusting for multiple comparisons; 75 of these CpG loci had mean β differences of at least 0.2, making the differential methylation at these loci robust. Further adjustment for age and sex identified 26 differentially methylated CpG sites in 22 genes. Six of these genes exhibited hypermethylation, while 16 genes showed hypomethylation in melanomas compared with nevi. Each of these CpG loci had high sensitivity and specificity for melanoma diagnosis, with AUCs ranging from 0.84 to 1.0. In PAM analyses, 12 CpG sites in nine genes provided the most accurate discrimination of melanomas from nevi, with no misclassification.

Of the 22 genes identified, 14 (with 16 CpG loci) were significantly differentially methylated in an independent data set including dysplastic nevi after adjustment for age, sex, and multiple comparisons.

Only one other study has reported performance of the Illumina Cancer Panel I array on formalin-fixed compared with non-fixed tissues. In this study, comparison between matched FFPE and frozen surgical pathology replicates of follicular lymphomas and follicular hyperplasias showed high correlation (r^2^ > 0.95) (Killian et al., 2009), similar to our results using fresh and FFPE prepared melanoma cell lines. We provide additional data regarding array performance by performing a ‘dose–response curve’ for detection of tumor DNA methylation in the presence of non-tumor DNA, and we identify 70% tumor DNA as a lower limit of purity to obtain profiles representative of the target DNA. We conclude that samples with higher percentages non-melanocytic tissue components should be enriched to this level by selective procurement of tumor cells using manual or laser capture microdissection before array profiling.

A few of the genes identified in our study as hypermethylated in melanomas relative to nevi have previously been examined for promoter methylation in melanocytic lesions. Among our panel of 26 CpG loci, Muthusamy et al. (2006) were the first to report that *COL1A2* was methylated in 80% of melanomas. Subsequently, Koga et al. (2009) reported that *COL1A2* was hypermethylated in 50% and 69% of early and advanced stage melanomas, respectively, but not in nevi or normal skin. From our marker panel of 75 CpG loci with β-value differences of ≥0.2, Liu et al. (2008) found that *TNFSF10D* and *LOX* were methylated in 80% and 50%, respectively, of melanomas.

Interestingly, among our panel of 26 markers, 15 of 19 CpG loci exhibiting relative hypomethylation in melanomas showed uniformly high and nearly complete methylation in nevi. For example, three CpG loci in the transcription factor *RUNX3* showed nearly complete methylation in nevi. *RUNX3* was previously reported to be hypermethylated in 23% of melanoma cell lines (Furuta et al., 2004) but only infrequently in 4% of primary melanomas (Kitago et al., 2009), but nevi were not examined in this study, making it unclear whether *RUNX3* methylation levels were relatively increased or decreased with the acquisition of malignancy. *RUNX3* has been considered a tumor suppressor gene, exhibiting both hypermethylation and reduced expression in gastric and colon cancers (Subramaniam et al., 2009). However, recent studies suggest that *RUNX3* may have both tumor suppressor and oncogenic properties depending on the cellular context (Chuang and Ito, 2010) and may function as an oncogene in basal cell carcinomas (Salto-Tellez et al., 2006) and head and neck cancers (Tsunematsu et al., 2009).

Among our panel of 75 markers, *SYK* was found to be less methylated in melanomas compared with nevi. Muthusamy et al. (2006) first reported *SYK* to be methylated in 30% of melanoma tumors, and a subsequent study confirmed this finding (Liu et al., 2008). These previous results are not inconsistent with our study because we also found melanomas to be partially methylated; however, nevi were more uniformly and completely methylated. Several other published studies conducted array-based or comprehensive methylation screening of multiple loci in melanomas; however, the genes examined in these reports were not among those we found to exhibit differential methylation in melanomas compared with nevi (Guan et al., 2003; Tanemura et al., 2009; Jonsson et al., 2010; Bonazzi et al., 2009; reviewed in Schinke et al., 2010).

Among the 22 genes with CpG sites that distinguished melanomas from nevi, half possess immune-related functions, with several, including *ITK* and *LAT (*Andreotti et al., 2010)*, CD2* (Sewell et al., 1986), *TNFSF8* (Kennedy et al., 2006), *IFNG* (Lee et al., 2006b), and *IL2* (Kallies, 2008) operating in T-cell regulatory pathways. Other immune-related genes identified have reported functions in myeloid–myeloid cell interactions (*EMR3*) (Stacey et al., 2001), neutrophil microbicidal activity (*MPO*) (Arnhold and Flemmig, 2010), antigen recognition by T cells (*HLA-DPA1)* (Lombardi et al., 2001), innate immunity (*CARD15/NOD2*) (Strober et al., 2006), NF-κB activation (*TRIP6)* (Chastre et al., 2009; Li et al., 2005), or interferon pathways (*PSCA*) (Marra et al., 2010). Moreover, as a transcription factor, *RUNX3* is important in the development and maturation of immune cells, including T cells (Wong et al., 2010) and thus could be involved in the alterations in immune gene promoter methylation observed between melanomas and nevi. The discovery of altered methylation in T-cell regulatory and other immune genes in melanomas is not surprising because previous studies have shown that these tumors often stimulate a significant host immune response (Anichini et al., 2004; Hussein, 2005). Further study will be needed to determine whether the differentially methylated immune-related genes are expressed in melanoma cells or are localized to infiltrating lymphocytes.

Our tissue sets of melanomas included a spectrum of histologic types, including superficial spreading, nodular, lentigo maligna, acral lentiginous, spindle cell, and unclassifiable melanomas, indicating that certain DNA-methylation differences may be consistent across subtypes. The melanomas also had a wide range of Breslow thicknesses and were from a variety of anatomic sites that would have received varying degrees of sun exposure. In sample set #1, the nevi tested were chosen to be unequivocally benign by standard histologic criteria, with only one displaying slight atypia. In an independent set of melanomas and nevi that included benign dysplastic nevi (sample set #2), many of the same genes remained significant for differential methylation.

Our nevus tissues did not include Spitz nevi or Spitzoid melanomas, which can be very challenging for pathologists to diagnose (Barnhill et al., 1999); therefore, it will be important to determine whether our putative melanoma-specific methylation loci also distinguish Spitz nevi from melanomas. In addition, patients with melanoma in this study were older (mean age 61 years, P < 0.0001) than patients with nevus (mean age 29 years), but to address possible confounding because of age, we controlled for patient's age and sex in the models used to identify CpG loci that distinguish melanomas.

Our work demonstrates technical feasibility for high-throughput DNA-methylation profiling of FFPE melanocytic specimens, as are typically prepared in diagnostic settings. Using this methodology, our study is the first to identify a multilocus DNA-methylation signature that is highly accurate for discriminating melanomas from benign nevi, indicating that DNA methylation holds promise for molecular diagnosis of melanocytic lesions. Future studies will work toward determining the generalizability of results to diverse malignant and benign melanocytic lesions, applicability to borderline lesions, and performance on specimens from a variety of medical practices and laboratories. Studies examining whether our results have implications toward the understanding of melanogenesis also seem warranted.

## Supporting information

**Figure S1.** Correlation curves showing the reproducibility and effects of formalin fixation and normal cell contamination on melanocytic methylation profiles obtained with the Illumina GoldenGate methylation array.

**Table S1.** Clinical and histologic characteristics of cutaneous melanocytic nevi and primary melanomas evaluated for DNA promoter methylation.

**Table S2.** One-hundred-sixty-eight CpG loci exhibiting significant promoter methylation differences between melanomas and benign nevi in sample set #1.
