# Supplementary material for: DNA-methylation profiling distinguishes malignant melanomas from benign nevi
Source: Pigment Cell Melanoma Res. 2011 Jan 21;24(2):352–60. doi: 10.1111/j.1755-148X.2011.00828.x (PMC3073305; doi:10.1111/j.1755-148X.2011.00828.x)
Supplement: Supplementary file 4 [file pcmr0024-0352-SD4.doc]

**Appendix S1**

**Materials and Methods**

**Study samples**

Primary formalin-fixed paraffin-embedded (FFPE) invasive cutaneous melanomas (n=22) and melanocytic nevi (n=29) were obtained from the University of North Carolina (UNC) Hospitals Pathology Archives for sample set #1.  Primary FFPE invasive cutaneous melanomas (n=26) and melanocytic nevi (n=29) were obtained from the UNC and University of New Mexico (UNM) Hospitals Pathology Archives for sample set #2. Age, sex, and anatomic site were abstracted from the pathology reports. Two nevus samples in sample set #1 and one melanoma in sample set #2 were excluded from analyses because they did not meet filtering criteria due to more than 25% missing β-values. The clinical characteristics of the specimens that passed filtering criteria and were included in the analyses are shown in Table S1. Peripheral blood leukocytes and FFPE normal skin samples were obtained from the UNC Tissue Procurement Facility. Melanoma cell lines were obtained as previously described (Kaufma*n*n et al., 2008). The UNC and UNM Institutional Review Boards approved the study.

**Standardized pathology review and enrichment of melanoma or nevi**

Five µm-thick tissue sections were cut from each block containing melanoma or nevus and were mounted on uncoated glass slides. A hematoxylin and eosin (H&E) slide of each melanoma or nevus specimen was reviewed by an expert dermatopathologist (PG) to confirm diagnosis, classify histologic subtype, and score standard histopathology features. The dermatopathologist encircled the tumors on the H&E slides to use as guides for manual dissection of melanoma or nevus cells away from surrounding normal skin from tissue sections to achieve an estimated greater than 70% tumor purity.

**Cell lines and peripheral blood leukocytes**

Cell lines were grown in RPMI medium with 10% fetal bovine serum and 1% penicillin/streptomycin and harvested while in log growth phase. Cells were pelleted and divided into two portions. One portion was used for DNA extraction (non-fixed) and the other pellet was fixed in buffered formalin, embedded in paraffin, and sections were cut from the paraffin blocks and mounted on uncoated glass slides. DNAs obtained from PBLs and the Mel-505 cell line were mixed in varying proportions and used to evaluate the effect of contamination of the methylation profile of the Mel-505 melanoma cell line by ‘non-melanocytic’ PBL cells.

**DNA preparation**

DNA was prepared from FFPE nevi, melanoma, normal skin tissues, or cell line pellets as previously published (Thomas et al., 2004; Thomas et al., 2007). DNA was purified from non-fixed cell lines and PBLs using the FlexiGene DNA kit (Qiagen, Valencia, CA, USA).

**Bisulfite treatment of DNA**

Sodium bisulfite conversion of DNA (obtained from FFPE or non-fixed cells) was performed using the EZ DNA Methylation Gold kit (Zymo Research, Orange, CA, USA). Approximately 500-1000 ng DNA from each tissue specimen was mixed with 130 μl of CT Conversion Reagent in a PCR tube and cycled in a thermal cycler at 98^o^C for 10 minutes, 64^o^C for 2.5 hours, and stored at 4^o^C for up to 20 hours. The sample was then mixed with 600 μl M-binding buffer and spun through the Zymo-Spin IC column for 30 seconds (>10,000 x g). The column was washed with 100 μl of M-Wash buffer, spun, and incubated in 200 μl of M-Desulphonation buffer for 15-20 minutes. The column was then spun for 30 seconds (at >10,000 x g), washed twice with 200 μl M-Wash buffer, and spun at top speed. The sample was eluted from the column with 10μl M-Elution buffer and stored in a -20^o^C freezer prior to use in the Illumina Methylation assay. After bisulfite treatment, DNA quantity and concentration were measured by a Nanodrop spectrophotometer, and DNA concentration adjusted to 50-60 ng/μl.

**Illumina GoldenGate Cancer Panel I methylation array analysis**

Array-based DNA methylation profiling was accomplished using the Illumina GoldenGate Cancer Panel I methylation bead array (Illumina, San Diego, CA, USA) to simultaneously interrogate 1505 CpG loci associated with 807 cancer-related genes. The Illumina GoldenGate methylation assay was performed as described previously (Bibikova et al., 2006). Two allele-specific oligonucleotides (ASO) and 1 locus-specific oligo (LSO) are designed to interrogate each CpG site, with the LSO containing a sequence that corresponds to a specific address on the BeadArray. Bisulfite-converted DNAs were biotinylated and bound to paramagnetic particles, hybridized to ASO and LSO probes, and the hybridized ASO oligos were extended in a methylation-specific fashion, then ligated to the LSO probe to create amplifiable templates. The joining of two fragments to create a PCR template provides an added level of locus specificity. The PCR that followed used 2 fluorescently-labeled (Cy3, Cy5) and biotinylated universal PCR primers corresponding to the ASO sequences (P1, P2) and a common P3 primer that binds to the LSO sequence. Labeled amplicons were bound to paramagnetic particles and denatured, then, after filtering out the biotinylated strands, the fluor-labeled strands were hybridized to the Sentrix BeadArray under a temperature gradient and imaged using the BeadArray Scanner. Methylation status of the interrogated CpG sites was determined by comparing the ratio of the fluorescent signal from the methylated allele to the sum from the fluorescent signals of both methylated and unmethylated alleles. Controls for methylation status used on each bead array included the Zymo Universal Methylated DNA Standard as the positive, fully-methylated control, and a GenomePlex (Sigma) whole genome amplified (WGA) DNA used as the negative, unmethylated control. The methylation array data can be accessed through Gene Expression Omnibus.

**Bioinformatics and statistical analysis**

The data were assembled using the GenomeStudio Methylation software from Illumina. All array data points were represented by fluorescent signals from both methylated (Cy5) and unmethylated (Cy3) alleles. Background intensity computed from the negative control was subtracted from each data point. The methylation level of individual interrogated CpG sites was determined by the β-value, defined as the ratio of fluorescent signal from the methylated allele to the sum of the fluorescent signals of both the methylated and unmethylated alleles and calculated as β = max(Cy5,0)/(|Cy5|+|Cy3|+100). β-values ranged from 0 in the case of completely unmethylated to 1 in the case of fully methylated DNA.

The GenomeStudio Methylation Module software (Illumina) was used to create scatter plots to examine the relationship between cell line replicates and between FFPE and non-fixed samples. The correlation coefficient, r^2^, was calculated for each comparison. For studies of melanomas and nevi, average methylation β-values were derived from the multiple β-values calculated for each CpG site within the melanoma or nevus groups. Prior to clustering or further statistical analysis, filtering was performed to remove a total of 478 probes that corresponded to 68 CpG sites on the X chromosome and 410 that were reported to contain a single nucleotide polymorphism or repeat within the recognition sequence thus making the probes unreliable in at least some samples (Byun et al., 2009). In addition, a detection p-value computed by GenomeStudio and representing the probability that the signal from a given CpG locus is distinguishable from the negative controls was used as a metric for quality control for sample performance. β-values with a detection p-value greater than 10^−5^ were considered unreliable and set to be missing (Marsit et al., 2009). In sample set #1, two nevus samples with more than 25% missing β-values and 39 CpG loci with more than 20% missing samples were excluded from analysis. The final data contained 988 CpG loci in 646 genes and 49 samples (22 melanomas and 27 moles). In sample set #2, one melanoma sample with more than 25% missing β-values and 35 CpG loci with more than 20% missing samples were excluded from analysis (25 melanomas and 29 moles).

All subsequent statistical analyses were carried out using the R (<http://www.r-project.org/>) and SAS software. For exploratory/visualization purposes, unsupervised hierarchical clustering using the Euclidean metric and complete linkage was performed. To adjust for age or gender effect, a linear model was fitted to the logit transformed β-values using age and gender as covariates in comparing the methylation levels between melanomas and moles at each locus. Bonferroni correction was used to adjust for multiple comparisons, i.e., significant loci were selected with p-value ≤ 0.05/988 = 5.06×10^−5^, with an additional filter of mean adjusted β-value difference ≥ 0.2 between melanomas and moles to be clinically significant. In addition, the area under the receiver operating characteristics curve (AUC) was computed to summarize the accuracy of correctly classifying melanomas and moles using these significant loci.

The Prediction Analysis of Microarrays (PAM) approach (Tibshirani et al., 2002) was carried out to assess the classification of melanoma and nevus samples by the method of nearest shrunken centroids.

**Gene ontology analysis**

The DAVID Bioinformatics Resources 6.7 Functional Annotation Tool (<http://david.abcc.ncifcrf.gov/home.jsp>) was used to perform gene-GO term enrichment analysis to identify the most relevant GO terms associated with the 75 genes found to be differentially methylated between nevi and malignant melanomas.

**References**

Thomas, N.E., Alexander, A., Edmiston, S.N., [Parrish, E](http://www.ncbi.nlm.nih.gov/pubmed?term="Parrish E"%5BAuthor%5D)., [Millikan, R.C](http://www.ncbi.nlm.nih.gov/pubmed?term="Millikan RC"%5BAuthor%5D)., [Berwick, M](http://www.ncbi.nlm.nih.gov/pubmed?term="Berwick M"%5BAuthor%5D)., [Groben, P](http://www.ncbi.nlm.nih.gov/pubmed?term="Groben P"%5BAuthor%5D)., [Ollila, D.W](http://www.ncbi.nlm.nih.gov/pubmed?term="Ollila DW"%5BAuthor%5D)., [Mattingly, D](http://www.ncbi.nlm.nih.gov/pubmed?term="Mattingly D"%5BAuthor%5D)., and [Conway, K](http://www.ncbi.nlm.nih.gov/pubmed?term="Conway K"%5BAuthor%5D). (2004). Tandem BRAF mutations in primary invasive melanomas. J. Invest. Dermatol. *122*, 1245-1250.

Thomas, N.E., Edmiston, S.N., Alexander, A., [Millikan, R.C](http://www.ncbi.nlm.nih.gov/pubmed?term="Millikan RC"%5BAuthor%5D)., [Groben, P.A](http://www.ncbi.nlm.nih.gov/pubmed?term="Groben PA"%5BAuthor%5D)., [Hao, H](http://www.ncbi.nlm.nih.gov/pubmed?term="Hao H"%5BAuthor%5D)., [Tolbert, D](http://www.ncbi.nlm.nih.gov/pubmed?term="Tolbert D"%5BAuthor%5D)., [Berwick, M](http://www.ncbi.nlm.nih.gov/pubmed?term="Berwick M"%5BAuthor%5D)., [Busam, K](http://www.ncbi.nlm.nih.gov/pubmed?term="Busam K"%5BAuthor%5D)., [Begg, C.B](http://www.ncbi.nlm.nih.gov/pubmed?term="Begg CB"%5BAuthor%5D)., et al. (2007). Number of nevi and early-life ambient UV exposure are associated with BRAF-mutant melanoma. Cancer Epidemiol. Biomarkers Prev. *16*, 991-977.
